# Development and validation of a questionnaire for assessing parents’ health literacy regarding vision screening for children: a Delphi study

**DOI:** 10.1038/s41598-023-41006-7

**Published:** 2023-08-24

**Authors:** Ahuva Ravid-Saffir, Shulamit Sella, Hadas Ben-Eli

**Affiliations:** 1 Department of Optometry and Vision Science, Hadassah Academic College, Jerusalem, Israel; 2 Goldschleger Eye Institute, Sheba Medical Center, Tel-Hashomer, Ramat-Gan, Israel; 3 Department of Ophthalmology, Hadassah-Hebrew University Medical Center, Jerusalem, Israel

**Keywords:** Eye diseases, Health care

## Abstract

Preschool vision screening is recommended to reduce the incidence of amblyopia that persists into adulthood. However, parent’s perceptions regarding the importance of screening and early intervention may constitute a significant barrier to seeking vision exams and pursuing treatment. The aim of this study is to develop and validate a questionnaire for assessing parent’s awareness, perception and health literacy of children's vision tests. The questionnaire was developed using the Delphi method with experts from the fields of pediatric ophthalmology, optometry, orthoptics, pediatric medicine, social sciences and Mother and Child Health Care centers. Experts were provided with drafts of the questions iteratively in three rounds until a consensus was reached independently on the relevant items, coherently language and redundancies. For the first, second and third stages of the Delphi process, 17, 15 and 13 experts participated in the panel respectively. Validity was achieved by wide consensus among the panel on the relevance of each question, of 75%, 85% and 90%, for the three rounds respectively. Here we describe the final questionnaire, *EYES*: Evaluating Young-Children Eye health Survey, which includes 31 questions regarding demographics, ocular history, parental health literacy, and perceptions of vision and vison exams.

## Introduction

Preschool vision screening is a widely recommended practice that aims to reduce the incidence of amblyopia that persists into adulthood. Despite its importance, parents' perceptions regarding early screening and intervention may pose significant obstacles to seeking eye and vision examinations and pursuing necessary treatment^1^. Therefore, gaining an understanding of parents’ perceptions and knowledge regarding their children’s visual health may lead to effective interventions to improve adherence.

Visual development in childhood hinges on anatomical growth and exposure to visual stimuli^2,3^. Amblyopia, defined as partial or complete visual impairment resulting from impaired passage of visual stimulation from the eyes to the brain is^4^ characterized by reduced visual acuity (VA) in one or both eyes^5^. Amblyopia can lead to unilateral blindness in adults^6,7^, increases the risk of several binocular vision disorders^8^, and is commonly caused by factors like strabismus, refractive errors, or physical barriers such as cataract or ptosis^4,9,10^. Early intervention is essential in managing amblyopia^11,12^, with a 3% incidence rate in the population. However, undetected cases carry a significant risk of persistence into adulthood. Furthermore, uncorrected refractive errors in children, such as hyperopia, myopia, and astigmatism, negatively impact their academic and developmental progress, underscoring the need for timely detection and treatment^4,13–16^.

Studies have shown that moderate uncorrected hyperopia in preschoolers can be associated with a decrease in written literacy skills when compared with normal refractory status, according to research conducted by the Vision in Preschooler (VIP) group^17^. Additionally, uncorrected myopia has been found to be related to decreased visual attention skills, visual-motor function, and visual perception^17–19^. Uncorrected astigmatism is associated with reduced academic readiness in multiple developmental and educational domains among preschool-aged children^20^. These findings highlight the importance of early detection and correction of refractive errors in children to support their development.

Many healthcare organizations recommend vision screening tests for toddlers to detect and prevent visual impairments at an early stage. For instance, the American Association of Pediatric Ophthalmology and Strabismus (AAPOS) recommends three screening tests before the age of five^21^, while the U.S. Preventive Services Task Force (USPSTF) suggests at least one screening test between ages 3–5^22^. When performed effectively, vision screening can maximize the detection rate of problems and minimize the cost of referral^23^.

Despite the benefits of vision screening, it is only the initial step towards treatment, as the course of action depends on several factors, including parental adherence to referrals for comprehensive eye examinations. Unfortunately, parental adherence varies widely across countries, ranging from 25% in the USA^24–26^ to 83% in Germany^27^. In Israel, a recent study found that 54% of parents adhered to vision screening referrals, and barriers to adherence were primarily derived from the parents' perceptions of the importance of vision screening^1^.

Health literacy among parents plays a critical role in the success of vision screening and treatment for children. Numerous studies suggest that increasing parental health literacy can improve outcomes in vision screening and subsequent treatment^23,24,28,29^. Health literacy encompasses knowledge, motivation, and the ability of individuals to access, comprehend, and apply health information in their daily lives, including health services, disease prevention, and the promotion of health behaviors that maintain and enhance their quality of life^30^. To improve parental health literacy regarding vision screening and examinations, a survey of their awareness and perceptions is necessary.

Two attempts have been made to use the Delphi process to develop a questionnaire on visual health. However, these had only a small panel of experts, and did not specifically target parents of young children^31,32^. Thus, a valid questionnaire capable of evaluating parents' visual health literacy does not currently exist.

This study aims to use the Delphi process to fill this gap by developing and validating a questionnaire for assessing parent’s awareness, perception and knowledge of children's vision tests and willingness to adhere to recommendations.

## Methods

### Delphi process overview

**Figure 1:**
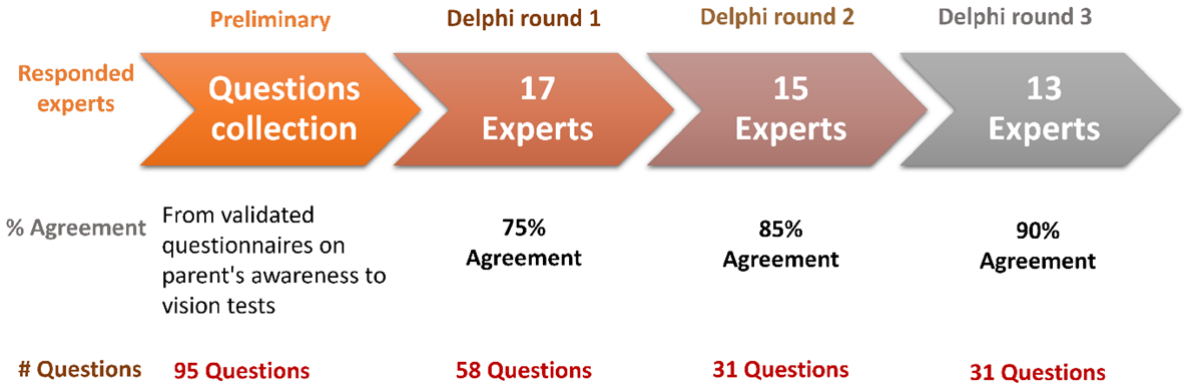
Delphi study stages are described graphically. The orange arrow on the left represents the preliminary questionnaire that was created based on validated questionnaires in the literature that assessed parent's visual health literacy. Each subsequent arrow represents the three Delphi rounds, with the number of experts who participated. The a priori threshold for agreement appears below each arrow.

The questionnaire was developed using the Delphi method (Fig. 1)^33,34^. The Delphi process is a widely used technique for achieving consensus among experts in various fields. It involves sending an initial questionnaire to experts from the field of interest, who respond anonymously and independently to each question, providing feedback and comments^35^. The process manager then drafts a revised questionnaire based on the initial responses, and several subsequent rounds are conducted until a consensus is achieved. This technique allows for the gathering of expert opinions without the need for a face-to-face meeting, and has been used in various fields, including healthcare research^33,34,36^. The current study follows recommendations for Delphi methodology in healthcare research^37^: identification of problem area of research, selection of panel, anonymity of panelists, controlled feedback, iterative Delphi rounds, consensus criteria, analysis of consensus, closing criteria, and stability of the results.

In the first step, questions collection was performed from the literature with questionnaires regarding parent's awareness of vision tests. Experts in children’s vision were provided with drafts of the questions iteratively, in three-rounds, until a consensus was reached independently on the relevant items that would be included in the questionnaire. Consensus was defined a priori as 75% of the experts agreeing on the inclusion of the questions. The experts were also asked to point out and comment on poor wording or redundancies in the proposed questions and to suggest missing questions. Validity was examined by calculating consensus between experts. The questionnaire was designed in English to allow participation of experts from abroad. After completion of the questionnaire, a translation of the final version into Hebrew was performed and examined by two different validators who confirmed that the translation was faithful to the original.

### Participants

The Delphi process included professionals who met the following criteria: optometrists with at least 10 years of experience, orthoptists licensed to practice orthoptics with at least three years of experience, pediatric ophthalmologists, preferably members of the Israel Society of Pediatric Ophthalmology, pediatricians, with priority given to physicians treating children in community clinics, social scientists, with at least a master’s degree, with a preference to those engaged in research and writing questionnaires, and past or present Mother and Child Health Care (MCHC) centers nurses, with at least three years of experience in the last decade.

### Question collection

Questions collection was done by a literature search in PubMed on articles from the last 15 years, using the search terms “development of questionnaire”, “knowledge”, “parents’ awareness”, “vision screening” and “children” from any health field and not limited to vision^38^. Papers were included if they had questionnaires on parents’ awareness of the importance of vision examinations and/or awareness of visual problems in children. Both open and closed questions were included from questionnaires, in-depth interviews and focus groups.

### Delphi round one

The experts were contacted by phone or email, and then the questionnaire and a letter explaining the Delphi process and how to complete the questionnaire were sent by e-mail (Supplementary Table 1). The experts were asked to address each question separately and answer the following questions: whether the question is relevant or irrelevant on a dichotomous scale, whether the question was coherently or incoherently worded (also on a dichotomous scale), and whether they had recommendations for rephrasing the questions (open question). It was also possible to add comments next to each question, and it was also possible to offer additional questions and make general comments. The questionnaires were sent over a four-week period and answers were collected over two months, during which reminder was sent to the participants.

After collecting the experts’ answers, the agreement rate was calculated for each question, as well as the proportion of experts who thought that the question was worded coherently and their comments. Questions were included in the next round if at least 75% of the panel thought it was relevant.

### Delphi round two

The second round of the Delphi process included all the questions that reached consensus on the first round after revision based on the critique provided. Additional questions that were suggested by the panel members were added. The questionnaire was built online in Google Forms and was send again to the panel (Supplementary Table 2). The panel was asked to respond to the relevancy of each question on a 5-point Likert scale with 1 being not relevant and 5 being relevant. The experts were given the opportunity to write whether the new wording of the question was clear and understandable and gave suggestions for alternative wording. The questionnaires collection took six weeks, and during that time, one reminder was sent to the panel. The degree of agreement for each question in the final questionnaire was then examined, and all the drafting comments and general comments were summarized. Answers of 4 or 5 on the Likert scale for relevancy were defined as recommending inclusion. The agreement was the proportion of the panel who recommended including the question and defined a priori as 85%.

### Delphi round three

This round included all the questions that reached consensus on the second round after revisions based on the critique provided. For each question, the experts were given three response options: recommend including the question in the final questionnaire, recommend including the question with revisions or exclude the question. Those who recommended excluding the question were asked to give reasons for their objection. Consensus was defined a priori as 90% of the panel members agreeing on its inclusion in the final questionnaire. The questionnaire was sent only to the panel members who answered the questionnaire of round one and two. The third round took six weeks, with one reminder sent.

The final questionnaire was translated from English to Hebrew by a professional translator with a background in health. The original questionnaire (in English) and the translated questionnaire (in Hebrew) were sent to two different people, one with a background in optometry and the other without a background in the field of health, in order to confirm that the Hebrew translation is faithful to the original.

### Statistical analysis

In each Delphi round, the agreement rate of the experts on the relevance of the questions was examined by calculating proportion and mean. An initial cut-off level for consensus was set at 75% in the first round. At the second round the experts rated their degree of consent for each question on a scale of 1–5 (1 = the question does not fit the questionnaire, 5 = the question fits the questionnaire) with a score of 4 or 5 being considered consent. At this round, the level of agreement threshold was set at 85%. On the third round, the agreement threshold was set at 90%. The degree of agreement of the experts, and their comments and suggestions for improvement provided the developed questionnaire with both internal validity and content validity. The analysis was performed using SPSS software (IBM SPSS Statistics, Version 27.0, Chicago. Armonk, NY: IBM Corp).

### Ethical approval

This study was performed in accordance with the 1964 Declaration of Helsinki and received the approval of the IRB Ethics Committee of Hadassah Academic College. The members of the expert panel gave written and verbal consent and their identity was kept anonymous. Their answers in all rounds were coded according to a numerical code.

### Informed consent

Informed consent was obtained from all individual participants included in the study.

## Results

### Question collection

A total of 11 articles were found^38– 48^ in the literature search that contained questions relevant to the field of pediatric vision testing (Fig. 1). After collecting all the questions, those that concerned teenagers and adults were omitted, and similar questions that appeared in several studies were included only once.

#### Round one

The question bank included 95 questions of various types (open, multiple choice or yes/no, Supplementary Table 1). Question number 95 was a multiple-choice question with 15 answers and the panel could respond to each answer. The questionnaires were sent to 29 professionals that agreed to participate in the panel, of which 17 responded (Table 1). All participants were Israeli aside from an American optometrist and a British optometrist.

**Table 1:** Response rate of experts by Delphi round.

	Round one N = 29	Round two N = 17	Round three N = 15
	Responded N (%)	Responded N (%)	Responded N (%)
Optometry	7 (24.1)	6 (35.3)	5 (33.3)
Social sciences	3 (10.3)	3 (17.6)	3 (20.0)
Pediatric ophthalmology	2 (6.9)	2 (11.8)	2 (13.3)
Orthoptics	2 (6.9)	2 (11.8)	2 (13.3)
Mother child health care nurses	2 (6.9)	1 (5.9)	1 (6.7)
Pediatric medicine	1 (3.1)	1 (5.9)	0 (0.0)
Total response rate	17 (58.6)	15 (88.2)	13 (86.7)
Didn’t respond	12 (41.4)	2 (11.8)	2 (13.3)

Out of 95 questions, 51 passed the screening threshold of 75% consensus, while 44 questions were rejected. When experts marked a question “incoherent” and provided critique, the requested changes were made in the question.

According to recommendation of the social scientists on the panel, open-ended questions (such as: “why did you apply for an eye test”) were omitted, though they received an overall high agreement rate. Three questions had high consensus but the panel suggested significant modification. Therefore, the original and modified versions were included in round two. Also, five new questions that were suggested by the panel were added to Delphi round two: “How many children do you have?”; “Do any of your child’s siblings wear glasses?”; “Has your child ever been examined by a pediatric ophthalmologist?”; “According to what you know, does your child have any eye problems?”; “When is it recommended for a child to undergo a visual examination?”.

### Round two

In round two, 58 questions were included in the questionnaire (Supplementary Table 3). The 17 professionals who responded in round one received a link to the online questionnaire, and 15 of them responded to it (13 responded to the online questionnaire and two on hard copy (Fig. 1 and Table 1)). Experts from all range of specialties were maintained in this round. Most of the comments that were received from the panel in round two were drafting comments and not on the content. Appropriate corrections were made.

For a few questions, a comment was received from the panel members that they are very similar and therefore redundant: “According to your knowledge does your child have any eye problems” and “according to your knowledge does your child currently have refractive error (e.g. near-sighted, far-sighted or astigmatism)”; “If your child has been asked to patch one eye, which eye is patched” and “If your child has been asked to patch one eye what is the purpose of the patching”. In these cases, the question with the highest score was included, and when the score for the questions was the same, the two versions were included in round three with an option to choose only one of them.

Two demographic questions on parent's education level and income received low agreement rate (76%) at this round. However, a review of previous studies^49,50^ demonstrated that education and income levels, though not directly related to the topic of vision screening, greatly impact knowledge and awareness. Thus, the demographic questions were retained for the third round.

Several of the panel members commented that it is inappropriate ask about religious affiliation. However, in many countries it is acceptable to ask about ethnic affiliation^51,52^. Furthermore, the Israeli Central Bureau of Statistics collects information on religious status. Thus, this question was included on the next round, offering the panel three possible versions.

In this round, three different questions dealing with the recommended times for vision tests were presented. Out of them, two questions received 86% consensus with a comment that they are very similar: “How frequently should a child receive a routine eye exam?”; When is it recommended for a child to undergo a visual examination?”. Therefore, the later was chosen for round 3. The third version of question received 90% consensus, but was phrased in such a way as to limit it only to parents whose child wears glasses: “If you obtained glasses for your child, how often should you bring your child for a routine vision check-up and prescription verification (In the absence of explicit instructions from the doctor)?” Thus, it was decided to retain the question that received a lower consensus rate but is intended for all respondents.

### Round three

Round three included 26 questions for review by the panel (16 questions for all parents and 10 additional questions for parents of children wearing glasses) and five demographic questions that were not sent to the panel. The questionnaire was sent online to the 15 experts who responded on round two, and 13 of these responded to it (11 responded to the online questionnaire and 2 and responded on hard copy; Fig. 1 and Table 1). Aside from the pediatrician, the experts maintained all specialties.

An a priori threshold of 90% agreement for each question was set at this round (12 out of 13 experts’ consensus), so that a question with two or more opponents was rejected. Out of 26 questions presented in this round, 13 questions received 100% approval, 7 questions received 92% approval (one opponent), while 6 questions received less than 90% approval (2 opponents or more) and were omitted from the final questionnaire. For the 20 questions that received more than 90% consensus, all the comments received were collected (both those comments that recommended inclusion and those comments that recommended disqualification), the comments were reviewed and corrected.

Several modifications were suggested on the questions that assess parents’ knowledge. Regarding multiple choice questions, there were comments suggesting to reduce the possible answers to questions. For questions with one correct answer, each question was then examined individually, since for some questions the large number of answers was due to a recommendation by panel members in a previous round. Regarding multiple choice questions with more than one correct answer, the answers were narrowed down without compromising the quality of the question. For one question that contained five sections, a number of comments were received claiming it was too complex, so it was broken down into five separate questions.

For three statements in the questionnaire a comment was received claimed that the statements were incorrect. A re-evaluation of these statements in the literature was done. For two statements: “Early treatment of vision problems is more effective” and “There are activities that can be harmful to vision”, it was found that a number of recent studies confirm the statements and therefore they have remained unchanged. For the third statement: “20 h a week of watching television does not impair vision”, a number of studies have found to support the claim, yet the maximum viewing time reported as vision harmless was over two hours a day (at least 14 h). Therefore, we adapted the statement to the questionnaire (14 weekly viewing hours instead of the 20).

### Final questionnaire

The final questionnaire was named the *EYES*: Evaluating Young-Children Eye health Survey and included thirty-one questions (Table 2). Of these seven are demographic: five are demographics of the parent and two child demographics. The parent is asked two questions regarding his or her ocular history and an additional 7 questions regarding their child’s ocular history. Parental health literacy of vision and vision exams is assessed with 7 questions and perceptions and attitudes regarding the importance of vision and vision exams by 8 questions. Twenty-seven questions (I-V, 1–22) are for parents of all children and the last 4 (23–26) for parents of children who were prescribed glasses.

**Table 2:** The final questionnaire—*EYES*: Evaluating young-children eye health survey.

#	Question	Type of question	Category
I	Sex (of parent) Female/Male	MCQ	Demographic parent
II	Age (of parent)	Open	Demographic parent
III	What is your religious affiliation? Muslim/Christian/Secular Jew/Orthodox Jew/Ultra-Orthodox Jew/Other	Closed question	Demographic parent
IV	What is the highest-level diploma or degree you received from your studies? Diploma from elementary school or junior high school/ Completed secondary, high school without a diploma or a matriculation certificate/ Matriculation certificate or secondary, high school diploma/ A post-secondary diploma that is not an academic degree (such as a teaching credential, practical engineering certificate, technician, nurse)/ Undergraduate academic degree, BA or equivalent degree MA, or an equivalent degree (including MD) Doctorate, PhD or equivalent degree/ Other:	Closed question	Demographic parent
V	Average monthly income per household is 10,000 NIS. What is your average monthly household income? Close to the average/Above average/Below average	Closed question	Demographic parent
1	Do you wear contact lenses or glasses (not reading glasses)?	Y/N question	Ocular history parent
2	Does someone in your family have eye problems before the age of six (such as lazy eye, wore glasses, crossed eyes, cataract)?	Y/N question	Ocular history parent
3	Treating eye problems before the age of 8 will have better outcomes than treating them later in life Strongly agree/agree/undecided/disagree/strongly disagree	Likert scale	Health literacy
4	Children’s vision only needs to be checked if child complains Strongly agree/agree/undecided/disagree/strongly disagree	Likert scale	Health literacy
5	How concerned would you be about your child’s eye health if: Someone in your family had strabismus (Crossed eyes) Not at all concerned—extremely concerned	Likert scale	Perceptions and attitudes
6	How concerned would you be about your child’s eye health if: one of the child’s grandparents had cataract Not at all concerned—extremely concerned	Likert scale	Perceptions and attitudes
7	How concerned would you be about your child’s eye health if: someone in your family had a high prescription Not at all concerned—extremely concerned	Likert scale	Perceptions and attitudes
8	How concerned would you be about your child’s eye health if: your child watches more than 14 h of TV per week Not at all concerned—extremely concerned	Likert scale	Perceptions and attitudes
9	How concerned would you be about your child’s eye health if: someone in your family had amblyopia (lazy eye) Not at all concerned—extremely concerned	Likert scale	Perceptions and attitudes
10	Some types of activities can aggravate eye problems Strongly agree/agree/undecided/disagree/strongly disagree	Likert scale	Health literacy
11	Which of the following do you think could be related to eye problems (can check more than one answer)? If a child frequently - Squints/Scratches eyes/Has ear pain/Has headaches/Has difficulty in school/Tilts their head	Multiple-choice question	Health literacy
12	How important is it to you that your child will be examined by an ophthalmologist? Very important/important/moderately important/ slightly important/ not important	Likert scale	Health literacy
13	When is it recommended for a child to undergo a visual examination? (Can have more than one answer) At 6–12 months/At age three/At age six/When there are complaints	Multiple-choice question	Health literacy
14	A child will complain when there is a visual problem Definitely/most probably/possibly/probably not/definitely not	Likert scale	Health literacy
15	Age of child (to which the survey refers)		Demographics child
16	Sex of child (to which the survey refers)	Closed question	Demographics child
17	Do any of your child’s siblings wear glasses?	Y/N question	Ocular history child
18	When was your child's first eye examination? At a mandatory screening (Tipat halav)/Before school enrollment/At first grade/Never/Other	Closed question	Ocular history child
19	Have you scheduled an appointment for an eye examination (for your child)?	Y/N question	Ocular history child
20	Were you informed of your child's eye examination tests results?	Y/N question	Ocular history child
21	Has your child ever been examined by an ophthalmologist?	Y/N question	Ocular history child
22	According to your knowledge, does your child have any eye problem (such as lazy eye, crossed eyes, need glasses, cataract)? Yes, my child has an eye problem/No, my child does not have eye problem/Don’t know	Y/N question	Ocular history child
23	Did you obtain eyeglasses for your child to correct their current vision problem? Not relevant/yes/no	Y/N question	Ocular history child
24	If you did not obtain glasses for your child, what is your main reason? I don’t want my child to wear glasses/There is no optical shop nearby/Too expensive/To prevent further increase in refractive error/Other	Closed question	Perceptions and attitudes
25	If your child does not wear eyeglasses most of the time, what is the main reason? Not necessary, can still see without eyeglasses/Cannot see even with eyeglasses/Not comfortable with eyeglasses/Eyeglasses will make the problem worse/Child doesn’t look good with eyeglasses/The doctor recommended only partial wear/Other	Closed question	Perceptions and attitudes
26	How often should you bring your child for a routine vision check-up or prescription verification (In the absence of explicit instructions from the doctor)? Less than 6 months/Six months to almost one year/One year/More than 1 year	Closed question	Perceptions and attitudes

## Discussion

In this study we report on the development of a 31-question survey that addresses a significant gap in the assessment of parental awareness, perception, and health literacy of children's vision tests, as well as their willingness to adhere to recommendations. The Delphi process was utilized in the construction of the questionnaire, with a panel of experts from the fields of vision, child-care, and social sciences providing input. Through a process of consensus-building, the questionnaire was refined, with experts encouraged to add, comment, and make suggestions for improvement.

There was broad agreement between experts that information on the importance of vision tests and signs and symptoms of vision problems as well as amblyogenic risk factors are of great importance. On the recommendation of the panel, the final questionnaire contains questions regarding family history and the recommended age for bringing children to vision tests. In contrast, consensus was not reached regarding the inclusion of questions on the topics of amblyopia, the differences between types of vision tests, and refractive impairment. Among these were definition of myopia and amblyopia, questions that distinguish between different types of vision tests (vision screening versus complete vision tests and tests to detect eye diseases) and various health care providers of the eyes (optometrist, general ophthalmologist and pediatric ophthalmologist), feasibility for the appropriate age for wearing glasses in childhood (about 60% in round one), congenital cataract (76% in round one, 65% in round two), strabismus treatment (less than 70% in round one) and amblyopia treatment (75% in round three). Questions regarding the parents' sources of information and barriers for seeking vision treatment were also removed.

During Delphi rounds of our study, a number of comments were received about the use of professional jargon and it was recommended to use lay terms familiar to the general population. These comments are in line with the conclusions of the research of Paasche-Orlow and colleagues who recommend using popular and simple terms rather than professional terms in questionnaires^53^.

Attention was paid in the development of this questionnaire to avoid built-in bias. In the Delphi process, a built-in risk of bias may be added by the types of preliminary questions submitted to the panel of experts^54^. To avoid this bias and for the questionnaire to be valid, the questionnaire must exhaust all the relevant aspects related to the subject under study^36,55^. Therefore, in the current study all the questions found in the relevant literature were used without exercising discretion. Thus, the initial questionnaire in the first round embodies the opinion of many researchers regarding all aspects of parental awareness and adherence to vision tests in children.

The current study involves the consensus of more experts than previous studies on the topic of parental awareness to their children's’ eye problems and diseases. Several studies have used Delphi method to validate questionnaire on the field of ophthalmology and eye diseases. Al-Lahim and colleagues developed a questionnaire regarding common eye diseases among the general population based on the opinion of two ophthalmologists and one family doctor^31^. Similarly, Megbelayin and Mboho in 2016 based their questionnaire on the same topic on four professionals: two ophthalmologists and two epidemiologists^32^. Another study that used Delphi method to validate a questionnaire assessing knowledge of diabetes and acceptance of eye care among people with diabetes, based the validation on consultation with six experts in the field and on focus groups^56^. Additionally, AbdulRahman and colleagues assessed the knowledge and practice of primary eye care among primary healthcare workers using modified Delphi technique on randomly eight workers in two rounds^57^. In contrast, in other fields such as antibiotic use, the researchers followed a structured Delphi process in which 18 different professionals participated in three rounds of consensus building^38^. Similarly, questionnaires have also been developed in the field of pain medicine^55^, mental health^58^ and quality of life^59^ using a wide panel of experts. The development of the questionnaire through a Delphi process provides consensus of a wide spectrum of expertise and is the main path to achieving validity. Thus, the current study with a starting panel of 17 experts, provides a much more valid questionnaire than previous attempts.

The years of seniority required for panel members differed by profession to reflect their exposure to pediatric vision. Israeli optometrists required more seniority due to the fact that they usually examine fewer children, since according to Israeli law they are only allowed to examine children under the supervision or referral of an ophthalmologist. Orthoptics and MCHC nurses, on the other hand, examine children routinely in their work routine. Pediatricians and pediatric ophthalmologists did not require additional seniority since they already examine many children while specializing in these professions.

Parental adherence to vision screening and to bringing children who fail to full exams is low worldwide^1,24,25,60^. In many vision screening programs, only 20–50% of the parents adhere the referrals, and the school-based eye care programs rarely referred failed children for long-term follow-up care needs^26^. A major barrier to seeking vision exams and treatment is the lack of knowledge about the importance of the tests and early treatment^1,61^. There is also a positive correlation between understanding the importance of vision tests in parents and taking children for treatment^62–64^. A study examining the effect of parents' knowledge on adherence to the instructions of their children's amblyopia treatment found that the main reasons for not adhering to the recommended treatment were lack of knowledge and understanding^64^. It was previously reported that educating parents, teachers and children regarding refractive errors and the importance of the correction with glasses can potentially increase spectacle wear amongst children^64^. Also, when parents were given information and an explanation about the importance of amblyopia care, there was an increase in the rate of responsiveness and adherence to treatment guidelines^64^. Similarly, educating parents during their children’s vision screening on five topics increased adherence to referrals: lazy eye, signs and symptoms of vision problems, refractory defects, difference between vision screening tests and comprehensive vision tests, and the importance of performing vision tests^63^.

The implementation of this questionnaire may lead to interventions that improve parental adherence to vision screening and exams. The results of this questionnaire can be used to design an educational program that will improve parents’ knowledge and the actual parental behavior. Through the newly developed questionnaire one can measure the "health education" of parents, identify populations with low knowledge and awareness and build a focused educational program to improve knowledge and awareness.

However, the questionnaire might need adaptation for use in other countries for cultural differences and specific healthcare systems. Since most of the experts are Israeli, their opinions might not be in line with opinions of experts from other health care systems. A specific example is the question on the recommended age for vision screening. This is at ages three or six in Israel as suggested by AAPOS^21^, but many European countries do this at age four^65^. The conditions targeted in any questionnaire should depend on their prevalence in the country. For example, in Israel there is a high prevalence of high myopia^66^ making this a target condition.

One of the limitations of this study is the need to narrow down definitions during the Delphi rounds, in a way that has omitted several issues. Thus, the questionnaire does not address the importance and manner of treating strabismus and amblyopia, although studies have shown that in these areas there is a direct and strong correlation between knowledge and awareness of responding to treatment. As a result of this limitation, it is possible that the developed questionnaire will have a reduced sensitivity for the analysis of the knowledge and awareness of parents of children diagnosed with strabismus and amblyopia and may even cause bias in the results of the general population. In addition, the panel of experts, which consisted mostly of Israeli professionals, recommended omitting issues related to the various types of vision exams and parents' sources of knowledge, as they believed that these issues were not relevant to the Israeli health system.

Other limitations involve the methodology used. A 5-point Likert scale was used in round 2, which allowed for neutral position. Finally, the final questionnaire is long and may prove burdensome for some parents. This will be evaluated in future studies.

In conclusion, a questionnaire was developed and validated for the first time in a developed country on parental health literacy, perceptions, awareness and behavior regarding their children's vision tests. This questionnaire can be used as a significant tool to analyze and understand the current status of parents’ awareness and adherence to vision tests and to develop targeted intervention programs.

In a future study the questionnaire will be deployed to parents and validated for statistical factor analysis and internal consistency.

### Supplementary Information

Supplementary Table 1.

Supplementary Table 2.

Supplementary Table 3.

## Data Availability

The datasets used and/or analyzed during the current study available from the corresponding author on reasonable request.
