# Supplementary material for: Development and validation of a questionnaire for assessing parents’ health literacy regarding vision screening for children: a Delphi study
Source: Sci Rep. 2023 Aug 24;13:13887. doi: 10.1038/s41598-023-41006-7 (PMC10449776; doi:10.1038/s41598-023-41006-7)
Supplement: Supplementary file 1 — Supplementary Table 1. [file 41598_2023_41006_MOESM1_ESM.pdf]

Supplementary table 1: Round 1 questionnaire with percent consensus. The references for the questions are provided in parentheses. Consensus was defined a priori as equal to or greater than 75%

	**Question + reference**	**Type of question**	**percent consensus**
1	Sex (male/ female) (Aldebasi ;2011, He. et al; 2005)	Multiple-Choice Question	94%
2	age (18-25, 26-35, 36 and up) (Islam. et al; 2015)	Multiple-Choice Question	94%
3	wears glasses or CL (not reading) yes/no (Aldebasi; 2011)	yes/no question	94%
4	parental education (primary/junior high/senior high/college) (He. et al; 2005 , Aldebasi ;2011)	Multiple-Choice Question	94%
5	Ethnicity (sector) (Kimel; 2006)	Multiple-Choice Question	88%
6	Socio-economic status (Islam. et al; 2015 , Kimel; 2006)	Multiple-Choice Question	88%
7	Did you participate in school vision screening as a child? (Yawn et al.; 1998)	yes/no question	65%
8	AGE of child (0-2.11, 3-5.12, 6-7.11) (He. et al; 2005)	Multiple-Choice Question	88%
9	Gender boy/girl (Kimel; 2006)	Multiple-Choice Question	88%
10	According to what you know, does your child currently have refractive error, such as nearsighted, farsighted, or astigmatism? a) No b) Yes c) Don't Know (He. et al; 2005)	Multiple-Choice Question	76%
11	Family history of child eye conditions (Fong et al. 2018)	yes/no question	88%

12	Length of ongoing patient care of child (Fong et al. 2018)	Open	59%
13	“How frequently should a child receive an eye exam? only when a problem arises / One time a year / Two times a year / Three times a year / Four times a year / Other (Ramai. et al.; 2015)	Multiple-Choice Question	82%
14	First vision screening:1. Before school enrollment 2. At a mandatory screening *(Al-Qesair & Alshammari; 2016)*	Multiple-Choice Question	82%
15	Health facility of taking first vision screening: family health center/ health maintenance organization / Private *(Al-Qesair & Alshammari; 2016)*	Multiple-Choice Question	71%
16	Family health center conducted vision screenings in the past 2 years: yes/no *(Al-Qesair & Alshammari; 2016)*	yes/no question	53%
17	Place of detecting a visual problem: A mandatory screening/ Periodic screenings/ A school /A family *(Al-Qesair & Alshammari; 2016)*	Multiple-Choice Question	71%
18	Did you obtain eyeglasses for your child to correct the current refractive error? a) No b) Yes (He. et al; 2005)	yes/no question	82%
19	If you did not obtain eyeglasses, what were the reasons? 1) Don’t want child to wear eyeglasses 2) No optic shop near by 3) Too expensive 4) Don’t know how to obtain eyeglasses 5) Other (He. et al; 2005)	Multiple-Choice Question	88%

20	If you obtained eyeglasses for your child, how often did you bring your child for a vision check-up and eyeglasses updating? 1) Less than 6 months 2) Six months to almost one year 3) One year 4) More than 1 year (He,et al; 2005)	Multiple-Choice Question	88%
21	If your child has eyeglasses, how frequent does he/she wear them? 1) Never 2) Sometimes 3) Most of time 4) Always (He,et al; 2005)	Multiple-Choice Question	94%
22	If your child does not wear eyeglasses most of time, what are the reasons? 1) Not necessary, can still see without eyeglasses 2) Cannot see even with eyeglasses 3) Not comfortable with eyeglasses 4) Eyeglasses will lead to progression 5) Child doesn't look good with eyeglasses 6) Other 7) Don't know (He. et al; 2005)	Multiple-Choice Question	94%
23	do you have knowledge of family health center location? *(Al-Qesair & Alshammari; 2016)*	yes/no question	53%
24	is there a difference between visual acuity & eye exam? Y/N don't know *(Al-Qesair & Alshammari; 2016)*	Multiple-Choice Question	71%
25	Is a visual acuity screening the same as an eye examination? (Kimel; 2006)	yes/no question	76%
26	Do you know what amblyopia is? (Kimel; 2006)	yes/no question	47%
27	Did you know that strabismus might lead to a loss of visual acuity in 1 eye? (Kimel; 2006)	yes/no question	65%

28	Can eyeglasses correct all eye problems? Y/ N / Don’t know (Kimel; 2006)	Multiple-Choice Question	71%
29	Did you know that treating eye problems before the age of 8 will have better outcomes than treating them later in life? (Kimel; 2006)	yes/no question	82%
30	What is the function of the lens? (Fong et al. 2018)	Open question	53%
31	What is the function of the retina? (Fong et al. 2018)	Open question	44%
32	What is the function of the optic nerve? (Fong et al. 2018)	Open question	44%
33	What does visual acuity mean? (Fong et al. 2018)	Open question	76%
34	What does it mean to have a visual acuity of 20/60? (Fong et al. 2018)	Open question	71%
35	If an eye is myopic (nearsighted), what does this mean? (Fong et al. 2018)	Open question	76%
36	If an eye is hyperopic (farsighted), what does this mean?(Fong et al. 2018)	Open question	76%
37	What is astigmatism? (Fong et al. 2018)	Open question	65%
38	Is there a difference between vision and the eye’s prescription? (Fong et al. 2018)	Open question	53%
39	What possible reasons are there for prescribing glasses to children? (Fong et al. 2018)	Open question	59%
40	If an eye is amblyopic, what does this mean? (Fong et al. 2018)	Open question	65%
41	What is strabismus? (Fong et al. 2018 , Kimel; 2006)	Open question	59%

42	If your child has been asked to patch one eye to improve vision, which eye is patched? (Fong et al. 2018)	Open question	82%
43	If your child has been asked to patch one eye to improve vision, what is the purpose of patching? (Fong et al. 2018)	Open question	82%
44	At what age should amblyopia be treated? (Fong et al. 2018)	Open question	59%
45	vision of children need to be checked only if there is a problem (Nirmalan. et al.; 2004)	Likert Scale	82%
46	vision of children need to be checked only if child complains (Nirmalan. et al.; 2004)	Likert Scale	82%
47	children are taken to a doctor only if traditional or alternative systems of treatment not beneficial (Nirmalan. et al.; 2004)	Likert Scale	47%
48	eye doctors need to be seen only for major eye problems (Nirmalan. et al.; 2004)	Likert Scale	71%
49	eye doctors need to be seen only if not cured by general physician (Nirmalan. et al.; 2004)	Likert Scale	76%
50	eye doctors need to be seen only for injuries (Nirmalan. et al.; 2004)	Likert Scale	71%
51	primary care centers treat only minor problems (Nirmalan. et al.; 2004)	Likert Scale	59%
52	strabismus is a disease (Nirmalan. et al.; 2004)	Likert Scale	59%
53	strabismus is not treatable (Nirmalan. et al.; 2004)	Likert Scale	71%
54	strabismus leads to vision loss in the eye (Nirmalan. et al.; 2004)	Likert Scale	71%

55	strabismus is a sign of good luck (Nirmalan. et al.; 2004)	Likert Scale	35%
56	strabismus is treatable (Nirmalan. et al.; 2004)	Likert Scale	71%
57	surgery is required to correct strabismus (Nirmalan. et al.; 2004)	Likert Scale	65%
58	treatment for strabismus is necessary (Nirmalan. et al.; 2004)	Likert Scale	71%
59	cataract can occur in children (Nirmalan. et al.; 2004)	Likert Scale	76%
60	cataract in children is treatable (Nirmalan. et al.; 2004)	Likert Scale	76%
61	surgery is required to treat cataract in children (Nirmalan. et al.; 2004)	Likert Scale	76%
62	cataract in children can cause vision loss (Nirmalan. et al.; 2004)	Likert Scale	76%
63	cataract in children is caused by nutritional deficiancy (Nirmalan. et al.; 2004)	Likert Scale	65%
64	children can have vision problems (Nirmalan. et al.; 2004)	Likert Scale	76%
65	children can wear spactacles (Nirmalan. et al.; 2004)	Likert Scale	71%
66	children below 4 years cannot wear spectacles (Nirmalan. et al.; 2004)	Likert Scale	59%
67	children should wear spectacles only after 5 years (Nirmalan. et al.; 2004)	Likert Scale	59%
68	children can see by 2 to 6 month of life (Nirmalan. et al.; 2004)	Likert Scale	65%
69	children can see only from 1 to 2 year of life (Nirmalan. et al.; 2004)	Likert Scale	65%

70	children can see by 3 year (Nirmalan. et al.; 2004)	Likert Scale	65%
71	vision can be improved by eating certain foods (Nirmalan. et al.; 2004)	Likert Scale	65%
72	children can have eye problems (Nirmalan. et al.; 2004)	Likert Scale	76%
73	Is there any type of activity which can aggravate eye problems? (Senthilkumar. et al.; 2013)	Likert Scale	82%
74	What do you think parents do (think) when they are notified that their child has failed vision screening? (Yawn et al.; 1998)	Open question	82%
75	Family health center eye services include: General eye exams/ Eye disease treatment/ Don't know *(Al-Qesair & Alshammari; 2016)*	Multiple-Choice Question	76%
76	sex of the child is considered to take the child for treatment (Nirmalan. et al.; 2004)	Likert Scale	41%
77	strabismus is congenital (Nirmalan. et al.; 2004)	Likert Scale	65%
78	are the following related to eye problems if a child frequently – squints, itches eyes, has headaches, has difficulty in school, and has ear pain? (Ramai. et al.; 2015)	Multiple-Choice Question	94%
79	vision is good if the child looks at objects moved in front of the eye (Nirmalan. et al.; 2004)	Likert Scale	53%
80	For what reasons (eye problems) do you seek care? (Senthilkumar. et al.; 2013)	Open question	94%
81	When do you think the eye problem could harm the child in a physical/ mental way? (Senthilkumar. et al.; 2013)	Open question	71%

82	Family health center conducted Sufficient Vision Screenings: yes/no *(Al-Qesair & Alshammari; 2016)*	yes/no question	75%
83	Family health center's eye services rate : Good /Fair/ Poor (Al-Qesair & Alshammari; 2016)	Likert Scale	81%
84	How important is it to you that you have an eye doctor? (Owsley. et al.; 2006)	Likert Scale	94%
85	How important is vision to you? (Owsley. et al.; 2006)	Likert Scale	82%
86	Do you think you have some control over the health of your eyes? (Owsley. et al.; 2006)	Likert Scale	94%
87	What were your experiences with your school vision screening? (Yawn et al.; 1998)	Likert Scale	71%
88	What has been your experience with vision screening in your children's school(s)? (Yawn et al.; 1998)	Likert Scale	76%
89	Did you receive an invitation for a vision screening test at the family health center *(Al-Qesair & Alshammari; 2016)*	Likert Scale	82%
90	Did you receive health education sessions? *(Al-Qesair & Alshammari; 2016)*	Likert Scale	81%
91	were you informed of your childs vision screening tests results? *(Al-Qesair & Alshammari; 2016)*	Likert Scale	94%
92	How far away from the eye doctor do you live? (Owsley. et al.; 2006)	Open question	69%
93	Do you feel you have a choice of eye doctors in your area? (Owsley. et al.; 2006)	Open question	88%

94	Main reason for not visiting family health center: Far distance/ Material weakness/ Human resources weakness/ Never heard of family health center *(Al-Qesair & Alshammari; 2016)*	Multiple-Choice Question	82%
95	Have you scheduled an appointment? Y/N If not, why? (Prompts of 15 possible answers listed below)	Multiple-Choice Question	88%
	1. You do not think it is necessary for a young child to see an eye specialist.		82%
	2. Your child has higher priorities (such as other health or behavioral conditions) than seeing an eye specialist		71%
	. 3. There are no eye specialists close to where you live.		76%
	4. You do not know which kind of eye specialists your child should see.		76%
	5. You may forget to schedule an appointment.		59%
	6. You do not know how to schedule an appointment with an eye specialist.		76%
	7. You have to miss work to take your child to see an eye specialist.		71%
	8. You do not have transportation to reach an eye clinic.		82%
	9. You could not afford the financial cost.		65%
	10. Your child has no health insurance to cover it.		59%
	11. You may forget to attend an appointment if the waiting time is long.		71%

	12. Family issues (such as illness, disabilities, conflicts, etc.)		71%
	13. Treated simple problems myself.		65%
	14. you don't feel comfortable with eye doctor/general medical doctor		71%
	15. your Child has no eye problems		71%
	(Su. et al.; 2013 , Ramai. et al.; 2015)		
