# Supplementary material for: Development and validation of a questionnaire for assessing parents’ health literacy regarding vision screening for children: a Delphi study
Source: Sci Rep. 2023 Aug 24;13:13887. doi: 10.1038/s41598-023-41006-7 (PMC10449776; doi:10.1038/s41598-023-41006-7)
Supplement: Supplementary file 2 — Supplementary Table 2. [file 41598_2023_41006_MOESM2_ESM.pdf]

Supplementary table 2. Round 2 questionnaire. Consensus was defined a priori as 85%.

	Question	Type of question	percent consensus
1	Gender (of parent) Female / Male	Closed question	73
2	Age (of parent)	Open question	93
3	What is your religious affiliation<br>Muslim / Christian / Secular Jew / Orthodox Jew / Ultra-Orthodox Jew / Other	Multiple-Choice Question	60
4	Years of education	Open question	100
5	Average monthly income per household is 10,000 NIS. what is your average monthly household income:<br>Close to the average / Above average / Below average	Multiple-Choice Question	73
6	Do you wear contact lenses or glasses (not reading glasses)?	Y/N question	100
7	Did someone in your family have eye problems before the age of six (such as amblyopia, refractive error, strabismus)?	Y/N question	100
8	How many children do you have?	Open question New question suggested in round 1	80
9	Age of child (to which the survey refers)	Open question	100
10	Gender of child (to which the survey refers) Male / Female	Closed question	93

11	Does any of your child's siblings wear glasses?	Y/N question New question suggested in round 1	100
12	When was your child's first vision screening?<br>At a mandatory screening (Tipat halav) / Before school enrollment / At first grade / Never	Multiple-Choice Question	93
13	Did you receive an invitation for a vision screening test at the family health center (Tipat Halav)<br>Yes / No / Don't remember	Closed question	80
14	What is your main reason for not attending vision screening test at family health center (Tipat halav)?<br>Accessibility problem / Low availability of appointments / I have no time / Did not receive an invitation / Other	Multiple-Choice Question	80
15	Did you receive health education sessions?	Y/N question	40
16	Were you informed of your child's vision screening tests results?	Y/N question	93
17	Has your child ever been examined by a pediatric ophthalmologist?	Y/N question New question suggested in round 1	93
18	What could be a reason to seek eye care?	Open question	93
19	According to what you know, does your child have any eye problem?	Y/N question	100

		New question suggested in round 1	
20	According to what you know, does your child currently have refractive error, i.e. nearsighted, farsighted, or astigmatism?	Y/N question	93
21	Did you obtain eyeglasses for your child to correct their current vision problem?	Y/N question	100
22	If you did not obtain glasses for your child, what is your main reason? I don't want my child to wear glasses / There is no optical shop nearby / Too expensive / Other	Multiple-Choice Question	100
23	If you obtained glasses for your child, how often should you bring your child for a routine vision check-up and prescription verification (In the absence of explicit instructions from the doctor)? Less than 6 months / Six months to almost one year / One year / More than 1 year	Multiple-Choice Question	93
24	If your child has eyeglasses, how frequently does he/she wear them? Never / Rarely / Sometimes / Often / Always	Likert Scale	86.6
25	If your child does not wear eyeglasses most of the time, what is the main reason? Not necessary, can still see without eyeglasses / Cannot see even with eyeglasses / Not comfortable with	Multiple-Choice Question	93

	eyeglasses / Eyeglasses will lead to progression / Child doesn't look good with eyeglasses / The doctor recommended only partial wear / Other		
26	Have you scheduled an appointment for an eye exam (for your child)?	Y/N question	100
27	If not, why?		86.6
	1. You do not think it is necessary for a young child to see an eye specialist.	Multiple-Choice Question	80
	2. Your child has higher priorities (such as other health or behavioral conditions) than seeing an eye specialist		80
	3. There are no eye specialists close to where you live.		73
	4. You do not know which kind of eye specialists your child should see.		66.6
	5. You have to miss work to take your child to see an eye specialist.		73
	6. You do not know how to schedule an appointment with an eye specialist.		66.6
	7. Your Child has no eye problems		60

	8. You do not have transportation to reach an eye clinic.		66.6
	9. You don't feel comfortable with eye doctor/general medical doctor		73
	10. Family issues (such as illness, disabilities, conflicts, etc.)		66.6
	11. You may forget to attend an appointment if the waiting time is long.		80
28	Do you feel you have a choice of eye doctors in your area?	Y/N question	86.6
29	Rate Mother Child Health Center (Tipat halav) eye services:	Likert Scale	86.6
30	Mother Child Health Center (Tipat-halav) conduct adequate vision screenings: strongly agree / agree / undecided / disagree / strongly disagree	Likert Scale	80
31	How frequently should a child receive a routine eye exam? Only when a problem arises / Once a year / Once every Two years / Before elementary school / Other	Multiple-Choice Question	86.6
32	Is a visual acuity screening the same as an eye examination?	Y/N question	86.6
33	Treating eye problems before the age of 8 will have better outcomes than treating them later in life.	Likert Scale	93

	strongly agree / agree / undecided / disagree / strongly disagree		
34	The term "visual acuity" refers to:<br>Glasses Prescription / The ability to identify small details at a certain distance compared to a person with normal vision / The farthest point from the eye at which images are clear / Don’t know	Multiple-Choice Question	66.6
35	If an eye is nearsighted (Myopic), it means that<br>Far objects seem blurry but near objects are clear. / It is harder to focus on near objects than on far objects / Don’t know	Multiple-Choice Question	80
36	If an eye is farsighted (hyperopic), it means that<br>Far objects seem blurry but near objects are clear. / It is harder to focus on near objects than on far objects / Don’t know	Multiple-Choice Question	80
37	If your child has been asked to patch one eye, which eye is patched?<br>The healthy eye / with the better vision<br>The eye with the problem / worst vision<br>Don’t know	Multiple-Choice Question	93
38	If your child has been asked to patch one eye , what is the purpose of patching?	Multiple-Choice Question	93

	To improve vision / To cure strabismus / To reduce the prescription / Don't know		
39	Vision of children needs to be checked only if there is a problem<br>strongly agree / agree / undecided / disagree / strongly disagree	Likert Scale	86.6
40	Vision of children needs to be checked only if child complains<br>strongly agree / agree / undecided / disagree / strongly disagree	Likert Scale	93
41	Eye problems that were not treated before the age of 8 might cause low vision or even blindness in the affected eye<br>strongly agree / agree / undecided / disagree / strongly disagree	Likert Scale<br>New question suggested in round 1	86.6
42.1	How concerned would you be about your Childs eye health if: Someone in your family had strabismus<br>not at all concerned - extremely concerned	Likert Scale<br>New question suggested in round 1	100
42.2	How concerned would you be about your Childs eye health if: One of the Childs grandparents had cataract.<br>not at all concerned - extremely concerned	Likert Scale<br>New question suggested in round 1	
42.3	How concerned would you be about your Childs eye health if: Someone in your family had a high prescription<br>not at all concerned - extremely concerned	Likert Scale<br>New question suggested in round 1	
42.4	How concerned would you be about your Childs eye health if: Your child watches more than 20 hours of TV per week	Likert Scale<br>New question suggested in round 1	

	not at all concerned - extremely concerned		
42.5	How concerned would you be about your Childs eye health if: Someone in your family had amblyopia<br>not at all concerned - extremely concerned	Likert Scale New question suggested in round 1	
43	Eye doctors need to be seen only if problem was not cured by general physician<br>strongly agree / agree / undecided / disagree / strongly disagree	Likert Scale	80
44	Cataracts can occur in children<br>definitely / most probably / possibly / probably not / definitely not	Likert Scale	80
45	Cataract in children is treatable<br>strongly agree / agree / undecided / disagree / strongly disagree	Likert Scale	66.6
46	Surgery is required to treat cataracts in children<br>definitely / most probably / possibly / probably not / definitely not	Likert Scale	73
47	Cataracts in children can cause vision loss<br>usually true / often true / ocasionally true / rarely true / usually not true	Likert Scale	73
48	Children can have vision problems<br>strongly agree / agree / undecided / disagree / strongly disagree	Likert Scale	80
49	Children can have eye problems<br>strongly agree / agree / undecided / disagree / strongly disagree	Likert Scale	73
50	Some types of activities can aggravate eye problems	Likert Scale	100

	strongly agree / agree / undecided / disagree / strongly disagree		
51	Mother Child Health Center (Tipat halav) eye services include: General eye exams / Vision screening tests / Eye disease treatment / Don't know	Closed question	80
52	Which of the following do you think could be related to eye problems (can check more than one answer)? if a child frequently - Squints<br>Itches eyes<br>Has ear pain<br>Has headaches<br>Has difficulty in school<br>Tilts their head	Multiple-Choice Question	100
53	How important is it to you that you have an eye doctor? very important / important / moderately important / slightly important / not important	Likert Scale	93
54	How important is vision to you? very important / important / moderately important / slightly important / not important	Likert Scale	80
55	Do you think you have some control over the health of your eyes? to a great extent / somewhat / very little / not at all	Likert Scale	86.6
56	When is it recommended for a child to undergo a visual examination?	Closed question	86.6

	At one year of age / At age three / At age six / All of the above / When there are complaints	New question suggested in round 1	
57	A child will complain when there is a visual impairment<br>definitely / most probably / possibly / probably not / definitely not	Likert Scale	93
58	What do you think parents should do when they are notified that their child has failed vision screening?<br>Nothing / repeat vision examination in six months / go to a comprehensive eye examination / go to the emergency room	Closed question	93
