# Supplementary material for: Development and validation of a questionnaire for assessing parents’ health literacy regarding vision screening for children: a Delphi study
Source: Sci Rep. 2023 Aug 24;13:13887. doi: 10.1038/s41598-023-41006-7 (PMC10449776; doi:10.1038/s41598-023-41006-7)
Supplement: Supplementary file 3 — Supplementary Table 3. [file 41598_2023_41006_MOESM3_ESM.pdf]

Supplementary table 3: Round 3 questionnaire. Consensus was defined a priori as 90%.

	Question	Type of question	percent consensus
I	Gender (of parent) Female / Male	Multiple-Choice Question	Not rated in round 3
II	Age (of parent)	Open question	Not rated in round 3
III	What is your religious affiliation<br>Muslim / Christian / Secular Jew / Orthodox Jew / Ultra Orthodox Jew / Other	Multiple-Choice Question	Not rated in round 3
IV	What is the highest level diploma or degree you received from your studies?<br>Diploma from elementary school or junior high school/ Completed secondary, high school without a diploma or a matriculation certificate/ matriculation certificate or secondary, high school diploma/ A post-secondary diploma that is not an academic degree (such as a teaching credential, practical engineering certificate, technician, nurse)/ Undergraduate academic degree, BA or equivalent degree/ MA, or an equivalent degree (including MD)/ Doctorate, PhD or equivalent degree/ Other:	Multiple-Choice Question	Not rated in round 3
V	Average monthly income per household is 10,000 NIS. what is your average monthly household income?<br>Close to the average<br>Above average	Multiple-Choice Question	Not rated in round 3

	Below average		
1	Do you wear contact lenses or glasses (not reading glasses)?	Y/N question	92
2	Did someone in your family have eye problems before the age of six (such as amblyopia, refractive error, strabismus)?	Y/N question	100
3	Treating eye problems before the age of 8 will have better outcomes than treating them later in life.<br>strongly agree / agree / undecided / disagree / strongly disagree	Likert Scale	92
4	If a child has been asked to patch one eye, which eye is patched?<br>The healthy eye / with the better vision<br>The eye with the problem / worst vision / Don't know	Multiple-Choice Question	77
5	If a child has been asked to patch one eye , what is the purpose of patching?<br>To improve vision / To cure strabismus / To reduce the prescription / Don't know	Multiple-Choice Question	77
6	Children's vision only needs to be checked if child complains<br>strongly agree / agree / undecided / disagree / strongly disagree	Likert Scale	100
7.1	How concerned would you be about your Childs eye health if: Someone in your family had strabismus<br>not at all concerned - extremely concerned	Likert Scale	100

7.2	How concerned would you be about your Childs eye health if: One of the Childs grandparents had cataract.<br>not at all concerned - extremely concerned	Likert Scale	
7.3	How concerned would you be about your Childs eye health if: Someone in your family had a high prescription<br>not at all concerned - extremely concerned	Likert Scale	
7.4	How concerned would you be about your Childs eye health if: Your child watches more than 20 hours of TV per week<br>not at all concerned - extremely concerned	Likert Scale	
7.5	How concerned would you be about your Childs eye health if: Someone in your family had amblyopia<br>not at all concerned - extremely concerned	Likert Scale	
8	Some types of activities can aggravate eye problems<br>strongly agree / agree / undecided / disagree / strongly disagree	Likert Scale	85
9	Which of the following do you think could be related to eye problems (can check more than one answer)? if a child frequently -<br>Squints / Itches eyes / Has ear pain / Has headaches / Has difficulty in school / Tilts their head	Multiple-Choice Question	100
10	How important is it to you that your child will be examined by an ophthalmologist?<br>very important / important / moderately important / slightly important / not important	Likert Scale	92
11	When is it recommended for a child to undergo a visual examination? (can have more than one answer)<br>At 6-12 months / At age three / At age six / When there are complaints	Multiple-Choice Question	92

12	A child will complain when there is a visual problem<br>definitely / most probably / possibly / probably not / definitely not	Likert Scale	92
13	What do you think parents should do when they are notified that their child has failed vision screening?<br>Nothing / repeat vision examination in six months / have the child receive a comprehensive eye examination / go to the emergency room	Closed question	77
14	Age of child (to which the survey refers)		100
15	Gender of child (to which the survey refers)	Closed question	92
16	Do any of your child's siblings wear glasses?	Y/N question	100
17	When was your child's first vision screening?<br>At a mandatory screening (Tipat halav) / Before school enrollment / At first grade / Never / Other	Closed question	100
18	Have you scheduled an appointment for an eye examination (for your child)?	Y/N question	85
19	Were you informed of your child's vision screening tests results?	Y/N question	100
20	Has your child ever been examined by an ophthalmologist?	Y/N question	100
21	According to your knowledge, does your child have any eye problem?	Y/N question	9/13

22	According to your knowledge, does your child currently have refractive error, i.e. nearsighted, farsighted, or astigmatism?	Y/N question	5/13
23	Did you obtain eyeglasses for your child to correct their current vision problem	Y/N question	100
24	If you did not obtain glasses for your child, what is your main reason?<br>I don't want my child to wear glasses / There is no optical shop nearby / Too expensive / To prevent further increase in refractive error / Other	Closed question	100
25	If your child does not wear eyeglasses most of the time, what is the main reason?<br>Not necessary, can still see without eyeglasses / Cannot see even with eyeglasses / Not comfortable with eyeglasses / Eyeglasses will make the problem worse / Child doesn't look good with eyeglasses / The doctor recommended only partial wear / Other	Closed question	100
26	How often should you bring your child for a routine vision check-up or prescription verification (In the absence of explicit instructions from the doctor)?<br>Less than 6 months / Six months to almost one year / One year / More than 1 year	Closed question	100
	If your child has been asked to patch one eye, which eye is patched?	The panel was asked which version to include or if they should	4 said to include the first version, 6 to include the second version and three to
	If your child has been asked to patch one eye , what is the purpose of patching?		

		exclude both	exclude both. Therefore, not included in the final questionnaire
